# Seroepidemiology of Aino Virus in Farmed and Free-Ranging Cervids in the Republic of Korea

**DOI:** 10.3389/fvets.2021.702978

**Published:** 2021-08-20

**Authors:** Jung-Yong Yeh, Yun Ji Ga

**Affiliations:** Department of Life Sciences, College of Life Sciences and Bioengineering, Incheon National University, Incheon, South Korea

**Keywords:** Aino virus, cervids, risk factor, seroprevalence, The Republic of Korea

## Abstract

Aino virus is an infectious, non-contagious, vector-borne agent that has been implicated in arthrogryposis-hydranencephaly syndrome in newborn cattle, sheep, and goats. Information about reservoirs and host animal species susceptible to Aino virus remains unclear. To further explore the role of cervids in Aino virus infection transmission, we investigated cervid sera to determine the prevalence of Aino virus-neutralizing antibodies and to identify factors correlated with antibody positivity. We screened cervid serum samples collected in the Republic of Korea to better understand infection patterns in this animal species. Overall, Aino virus infection was widespread; 75 of 716 (10.5%, 95% [95% CI] = 8.4–13.4) farmed-cervid serum samples collected from 292 herds contained antibodies to Aino virus. Serological evidence of Aino virus infection was demonstrated in 5 of 43 free-ranging cervids, accounting for a prevalence rate of ~11.6% (95% CI = 4.6–26.4). Our results revealed that age class and geographic location affected seroprevalence. The main risk factors associated with Aino virus seroprevalence were older age (> 2 years old, OR = 2.221, 95% CI = 1.209–4.079, *P* = 0.009 in adults), southern provinces (OR = 2.432, 95% CI = 1.445–4.093, *P* = 0.001), and western provinces (OR = 1.905, 95% CI = 1.041–3.488, *P* = 0.034). The results in this study suggest that cervid species might serve as important hosts for the transmission of Aino virus, highlighting the need for careful monitoring of Aino virus infections in cervids.

## Introduction

Aino virus is an arthropod-borne virus belonging to the Simbu group of the family *Peribunyaviridae*, genus *Orthobunyavirus*. Aino virus is an infectious, non-contagious, vector-borne agent that has been implicated in arthrogryposis-hydranencephaly syndrome in calves (1, 2). A range of skeletal and neurological abnormalities can be shown in newborn cattle, sheep, and goats infected with Aino virus during gestation. Hydranencephaly or arthrogryposis, unilateral cavitation in the cerebrum, severe hydranencephaly and arthrogryposis, microcephaly and cerebellar hypoplasia can be included in the symptoms of Aino virus. The type of abnormality may be associated with the time of infection of the fetus; early infection is related to hydranencephaly, while later infection is related to arthrogryposis (3–5). It is transmitted by blood-sucking midges and is distributed worldwide in temperate-to-tropical regions, including Asia and Australia (3, 4, 6–9).

In addition to Aino virus, several Simbu serogroup viruses are known to cause important diseases in livestock. Akabane virus infects a wide range of wild ruminants and livestock, including cattle, sheep, goats, horses and pigs (10, 11), causing abortion, stillbirth, and congenital abnormalities. Akabane virus is widely distributed across Africa, the Middle East, East Asia and Australia. From 1972 to 1975, a serious outbreak of Akabane disease in Japan caused more than 31,000 cases of abortion, stillbirth and congenital arthrogryposis and hydranencephaly (12). Recently, Peaton virus and Shamonda virus have also been associated with congenital abnormalities in ruminants in Japan and Israel (13–15). Natural genome segment reassortment has been shown to contribute significantly to the evolution of orthobunyaviruses, occurring between different viruses and between variants of the same virus type (16–18). M segments are more distantly related than L and S segments and appear to be derived by reassortment from other yet unidentified orthobunyaviruses. It has been reported that Australian Aino virus isolates are reassortants, deriving their L and S segments from Akabane virus and Peaton virus, respectively (19–21).

Although Aino virus infection in adult animals is asymptomatic, the virus or its specific neutralizing antibody has been found in populations of cattle, buffalo, sheep, goats, camels and deer in the western Pacific region from Japan to Australia (5–7, 9, 22–26). In a 2007 study of thoracic fluids from aborted calves, virus neutralization assays indicated positivity rates of 11% for Aino virus in the Republic of Korea (ROK) (25). It was also reported that the seropositivity rates of Aino virus in cattle and native goats (*Capra hircus*) were 4.5 and 13.3%, respectively (9, 27). In a previous study in sentinel calves, the seropositivity rates for Aino virus ranged from <14.1 to 33.2% (23). Although it has been accepted that Aino virus is endemic in the ROK, the epidemiology of Aino virus infection is poorly defined.

Aino virus is maintained in the environment by unknown means; presumably, it cycles between insect vectors, such as *Culicoides* biting midges and mosquitoes, and susceptible hosts. However, information about reservoirs and host animal species susceptible to Aino virus remains unclear. To further explore the role of cervids in Aino virus infection in the ROK, we analyzed cervid sera to determine the prevalence of Aino virus-neutralizing antibodies and to identify factors correlated with antibody positivity. Finally, we screened serum samples from cervids collected in the ROK for Aino virus-specific antibodies to better understand infection patterns in this animal species of Aino virus.

## Materials and Methods

### Sampling Design and Samples for Disease Monitoring

The target population in this study was free-ranging and farmed cervids. The necessary sample size to estimate prevalence was calculated using Epitools-Epidemiological Calculators (Ausvet, Canberra, Australia) based on methods described by Humphry et al. (28). A total of 657 animals were required to analyze the nationwide prevalence of antibody to Aino virus based on 5% desired precision, 95% confidence and 15% assumed true prevalence. The expected prevalence was determined according to previously reported serological data from cattle and native goats in the ROK (9, 23, 27). Herds and animals within such herds were chosen by a simple random sampling method in each province based on the government's national statistics according to the methods previously reported (29). Serum samples from cervids were obtained from two separate sources: (1) the serum bank of the National Foot-and-Mouth Disease (FMD) Surveillance Program maintained by the Foreign Animal Diseases Division of the National Veterinary Research and Quarantine Service (Anyang, the ROK) in 2011; and (2) free-ranging cervids rescued by local rescue parties or housed at wildlife rescue centers during the 2011 FMD epidemic in the ROK (30).

A total of 716 cervid blood samples were collected from 292 herds all over the territory of the ROK for the purpose of active surveillance of foreign animal diseases, including FMD, in 2011 in the ROK. An additional 43 cervid blood samples from animals [Korean water deer (*Hydropotes inermis argyropus*) and Siberian roe deer (*Capreolus pygargus*)] rescued by local rescue parties or housed at captured wildlife rescue centers were available for testing. Cervid blood collection and serum preparation were performed according to previously reported methods (29).

### Serologic Testing

A serum neutralization test (SNT) was carried out to confirm serological evidence of Aino virus infection. Samples were heated to 56°C for 30 min to inactivate complement factors. Vero cells (C-1586, American Type Culture Collection, Manassas, VA, USA) were maintained in alpha-minimum essential medium (Gibco, Grand Island, NY, USA) containing 5% fetal bovine serum and antimycotic antibiotics (Gibco). SNTs against Aino virus were performed in flat-bottomed 96-well plates. A 100 50% tissue culture infective dose (TCID_50_) of the Aino virus strain KSA9910 (VR64, Korea Veterinary Culture Collection, Anyang, ROK) was added to a volume of 50 μL in the test wells of a flat-bottomed microtiter plate and mixed with an equal volume of serum that had been serially diluted in tissue culture medium. After this initial incubation, the virus–serum sample dilutions were dispensed into 96-well microplates containing a monolayer of Vero cells and cultivated as previously described (25, 31, 32). The virus-specific cytopathic effect (CPE) in 50% of the wells was assessed and scored after incubation for 3–5 days using an inverted microscope (Olympus. Tokyo, Japan). Antibody titers were expressed as the reciprocal of the highest serum dilution at which CPEs were inhibited. A titer of 1:4 or greater was considered positive. The apparent prevalence rates were considered animal-level prevalence, defined as the proportion of SNT-positive animals out of the total number of animals tested in the study area, and the herd prevalence was defined as the proportion of SNT-positive herds out of the total number of tested herds in the area. A herd was classified as positive if at least one animal in the herd was SNT positive. To evaluate serological cross-reactivity, Aino virus-SNT positive serum samples were subjected to serum neutralization testing for Akabane virus, which is a related veterinary arbovirus and an endemic Simbu group virus in the ROK.

### Risk Factor Analysis

Signs typical of Aino virus infection were not observed in any of the studied animals. Risk factor information was obtained from (1) the Korea Animal Health Integrated System (Animal and Plant Quarantine Agency, Anyang, ROK); (2) veterinarians in wildlife rescue centers; and (3) animal owners using a questionnaire form according to the methods previously reported (29). We investigated associations between the seroprevalence and seropositivity risk factors such as herd population size, age class, species, and location. To establish age classes, animals were classified into three age groups based on tooth replacement and livestock owner questionnaires: juveniles (between 6 months and 1 year old), subadults (between 1 and 2 years old), and adults (> 2 years old).

The apparent prevalence and 95% confidence interval (CI) (33) of true prevalence were calculated using Epitools-Epidemiological Calculators (Ausvet, Canberra, Australia). A logistic regression model was used to identify potential risk factors associated with animal seropositivity. The following individual exposure variables were assessed in the univariable analysis: herd size, age class, species, and region. The variables in the univariable analysis were evaluated for pairwise collinearity or associations by Pearson's correlation coefficient or the chi-squared test for continuous or categorical variables, respectively. The strength of association was analyzed using odds ratios and 95% CIs. A *P* < 0.05 (typically ≤ 0.05) was considered statistically significant. Statistical analyses in this study were performed by using the using commercially available statistical software (SPSS Statistics for Windows, Version 25.0, IBM Corp., Armonk, NY, USA).

## Results

**Figure 1:**
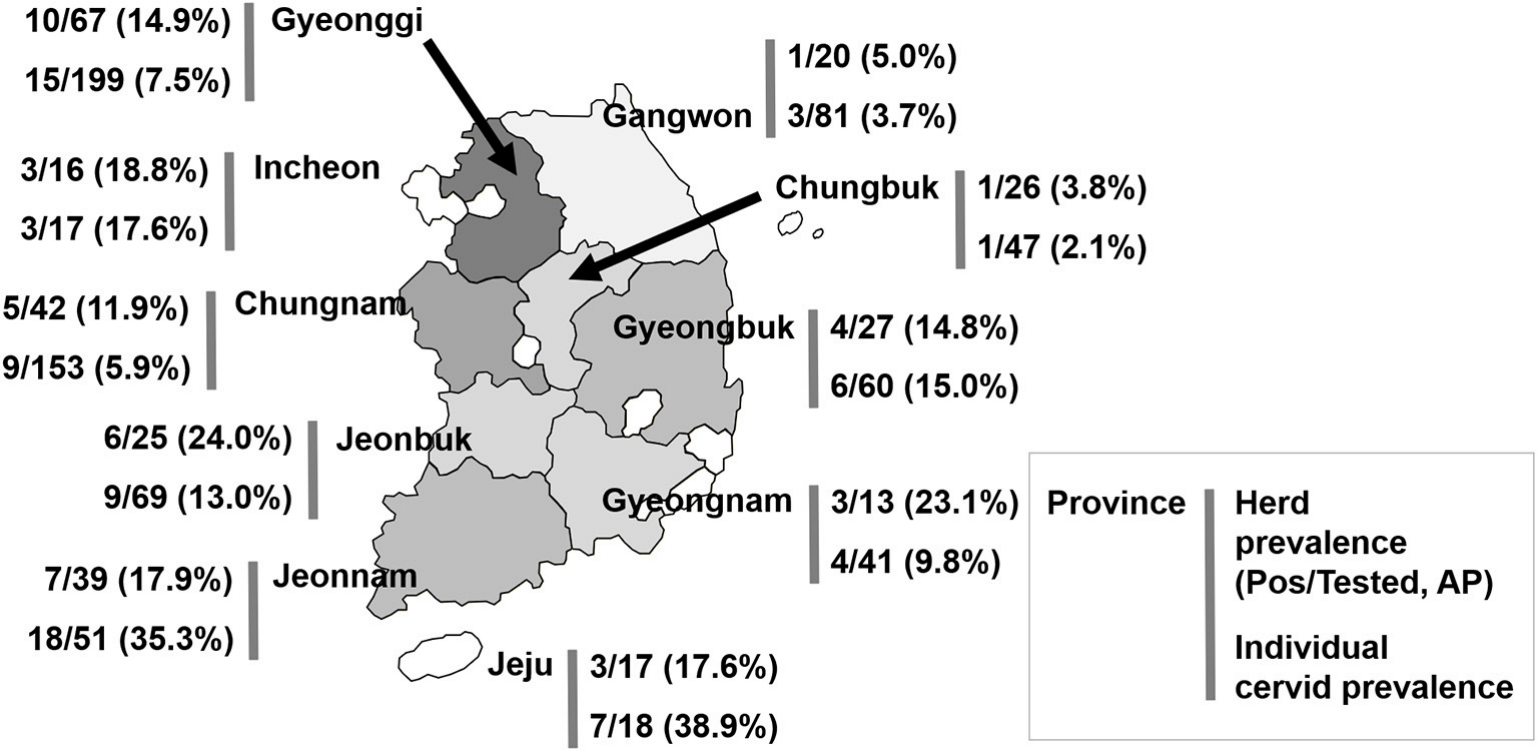
Geographical location of the provinces of the Republic of Korea and seroprevalence to Aino virus. AP, apparent (estimated) prevalence.

A total of 759 cervids were investigated in this study. Overall, Aino virus infection was widespread, as 75 of 716 (10.5%, 95% [95% CI] = 8.4–13.4) farmed cervid serum samples collected from 292 herds were shown to have antibodies to Aino virus as determined by SNTs. In addition, 43 herds analyzed in this study included seropositive animals (14.7%, 95% CI = 11.4–20.5), as demonstrated in Table 1. Seropositive cervids were identified in most provinces surveyed. The highest prevalence rates for Aino virus were shown in Jeonbuk Province, with 24.0% (95% CI = 11.8–47.7) of herds affected (6 out of 25), and Jeju Province, with 38.9% (95% CI = 25.4–54.0) of heads affected (18 out of 51), as seen in Figure 1.

**Table 1:** Seroprevalence of Aino virus in cervids in the Republic of Korea.

			**Herds**				**Individual cervids**			
**Province**	**Latitude (N)**	**Longitude (E)**	**Positive**	**Tested**	**AP**	**TP ± 95% CI**	**Positive**	**Tested**	**AP**	**TP ± 95% CI**
Incheon	36°55′−37°58′	124°36′−126°47′	3	16	18.8	6.3–47.2	3	17	17.6	5.8–45.0
Gyeonggi	36°53′−38°17′	126°22′−127°51′	10	67	14.9	8.2–27.4	15	199	7.5	4.1–12.4
Gangwon	38°09′−39°25′	126°46′−128°22′	1	20	5.0	9.0–23.6	3	81	3.7	0.0–10.5
Chungbuk	37°15′−36°00′	127°16′−128°38′	1	26	3.8	0.0–20.1	1	47	2.1	0.0–11.4
Chungnam	35°58′−37°03′	125°32′−127°38′	5	42	11.9	4.7–27.0	9	153	5.9	2.4–11.0
Jeonbuk	35°18′−36°09′	125°58′−127°54′	6	25	24.0	11.8–47.7	9	69	13.0	6.8–24.7
Jeonnam	33°54′−35°30′	125°04′−127°54′	7	39	17.9	9.0–35.6	18	51	35.3	25.4–54.0
Gyeongbuk	35°34′−37°33′	127°48′−131°52′	4	27	14.8	5.5–35.4	6	40	15.0	6.8–31.5
Gyeongnam	34°39′−35°54′	127°35′−129°28′	3	13	23.1	8.1–55.3	4	41	9.8	3.2–24.2
Jeju	33°06′−34°00′	126°08′−126°58′	3	17	17.6	5.8–45.0	7	18	38.9	21.7–67.8
Total	33°06′−39°25′	124°36′−131°52′	43	292	14.7	11.4–20.5	75	716	10.5	8.4–13.4

*AP, apparent (estimated) prevalence; TP, true prevalence; CI, confidence interval. A number of free-ranging animals, such as Korean water deer (Hydropotes inermis argyropus) and Siberian roe deer (Capreolus pygargus), were excluded because these animals were rescued by local rescue parties or captured wildlife rescue centers, and their herds could not be identified*.

Serological evidence of Aino virus infection was found in 5 of 43 free-ranging cervids that were rescued by local rescue parties or were housed at captured wildlife rescue centers, accounting for a serological prevalence of ~11.6% (95% CI = 4.6–26.4) (Table 2), as determined by SNTs. Free-ranging animals, such as Korean water deer (*Hydropotes inermis argyropus*) and Siberian roe deer (*Capreolus pygargus*), are excluded from Table 1 because the regionalities of the animals could not be determined in many cases.

**Table 2:** Univariable analysis of Aino virus exposure variables relative to seropositivity outcomes in cervid in the Republic of Korea.

**Variable**	**Positive**	**Negative**	**OR (95% CI)**	***P* value**
**Herd size**				
>50	15	193	1.830 (0.959–3.491)	0.064
10–50	29	230	1.622 (0.845–3.114)	0.143
<10	31	218	Reference	
**Age class**				
Juvenile	15	207	Reference	
Subadult	13	142	1.263 (0.583–2.736)	0.553
Adult	47	292	2.221 (1.209–4.079)	0.009
**Species**				
*Farmed*				
Elk (*Cervus canadensis*)	41	315	Reference	
Sika deer (*Cervus nippon*)	19	169	0.864 (0.486–1.535)	0.617
Red deer (*Cervus elaphus*)	15	157	0.734 (0.394–1.367)	0.328
*Free-ranging* ^†^				
Siberian roe deer (*Capreolus pygargus*)	2	17	0.904 (0.202–4.054)	0.895
Korean water deer (*Hydropotes inermis argyropus*)	3	21	1.098 (0.314–3.841)	0.884
Area^‡^				
Northern	22	322	Reference	
Southern	53	319	2.432 (1.445–4.093)	0.001
Eastern	14	195	Reference	
Western	61	446	1.905 (1.041–3.488)	0.034

*CI, confidence interval;*

† *Some blood samples in this study were sampled from animals rescued by local rescue parties or captured wildlife rescue centers because of car accidents or because their presence in the downtown area necessitated their removal. Number of these free-ranging animals such as Korean water deer (Hydropotes inermis argyropus) and Siberian roe deer (Capreolus pygargus) are included in free-ranging category;*

‡ *In this study, the northern area of the Republic of Korea includes Incheon, Gyeonggi, Gangwon, Chungbuk, and Chungnam provinces on the basis of latitude 36 degrees north whereas Southern area includes Ulsan, Jeonbuk, Jeonnam, Gyeongbuk, Gyeongnam, and Jeju. The western area includes Incheon, Gyeonggi, Chungnam, Jeonbuk, Jeonnam, and Jeju on the basis of longitude 127 degrees east, whereas the eastern area includes Ulsan, Gangwon, Gyeongbuk, and Gyeongnam*.

Our cervid serum sample analysis results collected in 2011 revealed that age class and geographic location affected seroprevalence. In the univariable analyses (Table 2), older age was shown to be a significant risk factor (OR = 2.221, 95% CI = 1.209–4.079, *P* = 0.009 in adults). The high seroprevalence in adult animals likely represents continuous episodes of transmission. Additionally, there were substantial regional differences in seroprevalence within the ROK. We observed a significant difference in the individual likelihood of positivity between southern provinces and northern provinces (OR = 2.432, 95% CI = 1.445–4.093, *P* = 0.001). We also observed a significant difference in the individual likelihood of positivity between western provinces and eastern provinces (OR = 1.905, 95% CI = 1.041–3.488, *P* = 0.034). Differences in seroprevalence between herd size and species were not significant (*P* > 0.05). All the Aino virus-SNT-positive cervid serum samples in this study were found to be serologically negative and did not cross-react with Akabane virus, which belongs to the Simbu serogroup of the genus *Orthobunyavirus*.

## Discussion

Aino virus seroprevalence was estimated to be 11.8% (farmed cervids 10.5% and free-ranging cervids 14.7%), demonstrating that exposure to this virus is prevalent among farmed and free-ranging cervids in the ROK despite the paucity of reported outbreaks, probably due to often unapparent clinical signs and underascertainment of Aino virus data.

Antibody cross-reactivity occurs among some arboviruses in the Simbu serogroup. Two newly emerged arboviruses, Peaton virus and Shamonda virus, have been classified into the Simbu serogroup of the genus *Orthobunyavirus*, similar to Akabane virus and Aino virus. Although Peaton virus and Shamonda virus were isolated sequentially in Japan, no Peaton or Shamonda virus infection cases have been reported in the ROK. Only one paper published in 2018 demonstrated serological evidence of Peaton and Shamonda viruses in a small number of animals (1.1 and 5.6%, respectively), without any clinical signs. Additionally, no cross-reactivity among Akabane virus, Aino virus, Peaton virus, and Shamonda virus was observed in neutralization tests (9, 34, 35).

In the present study, cross-reactivity with Akabane virus was not observed in cross-neutralization tests, suggesting that the antibody positivity against Aino virus observed in the present study was not due to a serological cross-reaction. This is in line with previous studies that found no serological cross-reactivity among Aino, Akabane, Peaton, and Shamonda viruses (9, 34–36), although the possibility that the antibodies are due to cross-reactivity with other Simbu serogroup viruses cannot be completely excluded.

For the first time, this report provides evidence of circulating antibodies against Aino virus among cervids in the ROK. Our estimate of the seroprevalence among cervids was lower than that found in Korean sentinel cattle in 2010 (33.2%) (23), native goats in 2005–2006 (13.3%) (24), and thoroughbred horses in 2007 (19.5%) (24).

The current study represents the first assessment of factors associated with Aino virus seropositivity in cervids in the ROK. The seroprevalence of Aino virus in cervids was significantly different among age cohorts and geographical locations. Aino virus prevalence among cervids in this study was highest in the older group, which may represent continuous transmission. Our results also demonstrated that the prevalence of Aino virus antibodies varied widely in the ROK. The reasons for the region-specific differences in Aino virus transmission are unclear, but the prevalence of Aino virus was higher in the southern and western regions of the ROK. One possibility is that there may be ecological and climatic factors that promote increased exposure to infected reservoirs in tropical rainforest regions. Alternatively, viral persistence or transmissibility may be higher in regions with elevated temperatures and rainfall.

## Conclusion

The results in this study demonstrated that Aino virus infection was prevalent among the farmed and free-ranging cervids surveyed; approximately one in 10 animals were infected with the virus. The present study demonstrates that Aino virus is widely distributed in the ROK and that although an outbreak of Aino virus among native cervids in the ROK has yet to be reported, susceptible cervids are at risk of becoming infected with Aino virus. The results in the present study suggest that cervid species might serve as important hosts for the transmission of Aino virus, highlighting the need for closer monitoring of Aino virus infections in cervids in the ROK. The results of this seroprevalence study may serve as a basis for future epidemiological studies of Aino virus infection in the ROK.

## Data Availability Statement

The original contributions presented in the study are included in the article/supplementary material, further inquiries can be directed to the corresponding author/s.

## Author Contributions

J-YY and YJG conducted the laboratory experiments and wrote the first draft. J-YY conceived and designed the study and revised the manuscript. All authors contributed to manuscript revision, read the manuscript, and approved the submitted version.

## Conflict of Interest

The authors declare that the research was conducted in the absence of any commercial or financial relationships that could be construed as a potential conflict of interest.

## Publisher's Note

All claims expressed in this article are solely those of the authors and do not necessarily represent those of their affiliated organizations, or those of the publisher, the editors and the reviewers. Any product that may be evaluated in this article, or claim that may be made by its manufacturer, is not guaranteed or endorsed by the publisher.
